# Supplementary material for: Fluid dynamics of COVID-19 airborne infection suggests urgent data for a scientific design of social distancing
Source: Sci Rep. 2020 Dec 30;10:22426. doi: 10.1038/s41598-020-80078-7 (PMC7773744; doi:10.1038/s41598-020-80078-7)
Supplement: Supplementary file 1 — Supplementary Information. [file 41598_2020_80078_MOESM1_ESM.pdf]

TABLE Supplementary 1. Physical/chemical parameters representative of expiratory events and adopted in the present investigation.

Mean ambient temperature	T	$25\,^{\circ}\mathrm{C}$
Crystallization (or efflorescence) RH	CRH	45%
Density of liquid water	ρ_w	$9.97 \times 10^2\,\mathrm{kg/m^3}$
Density of soluble aerosol part (NaCl)	ρ_s	$2.2 \times 10^3\,\mathrm{kg/m^3}$
Density of insoluble aerosol part (mucus)	ρ_u	$1.5 \times 10^3\,\mathrm{kg/m^3}$
Density of dry nucleus	ρ_N	$1.97 \times 10^3\,\mathrm{kg/m^3}$
Mass fraction of soluble material (NaCl) w.r.t. the total dry nucleus	ϵ_m	0.75
Mass fraction of dry nucleus w.r.t. the total droplet	$\mathcal{C}$	1 %
Specific gas constant of water vapor	R_v	$4.6 \times 10^2\,\mathrm{J/(kg\,K)}$
Diffusivity of water vapor	D_v	$2.5 \times 10^{-5}\,\mathrm{m^2/s}$
Density of air	ρ_a	$1.18\,\mathrm{kg/m^3}$
Kinematic viscosity of air	ν	$1.8 \times 10^{-5}\,\mathrm{m^2/s}$
Heat conductivity of dry air	k_a	$2.6 \times 10^{-2}\,\mathrm{W/K\,m}$
Latent heat for evaporation of liquid water	L_w	$2.3 \times 10^6\,\mathrm{J/kg}$
Saturation vapor pressure	e_{sat}	$0.616\,\mathrm{kPa}$
Droplet condensational growth rate	C_R	$1.5 \times 10^{-10}\,\mathrm{m^2/s}$
Surface tension between moist air and salty water	σ_w	$7.6 \times 10^{-2}\,\mathrm{J/m^2}$
Molar mass of NaCl	M_s	$5.9 \times 10^{-2}\,\mathrm{kg/mol}$
Molar mass of water	M_w	$1.8 \times 10^{-2}\,\mathrm{kg/mol}$
Vertical distance of the mouth opening from the floor	y_{mouth}	$1.6\,\mathrm{m}$
Cross-sectional area of the mouth opening	A_{mouth}	$4.5\,\mathrm{cm^2}$

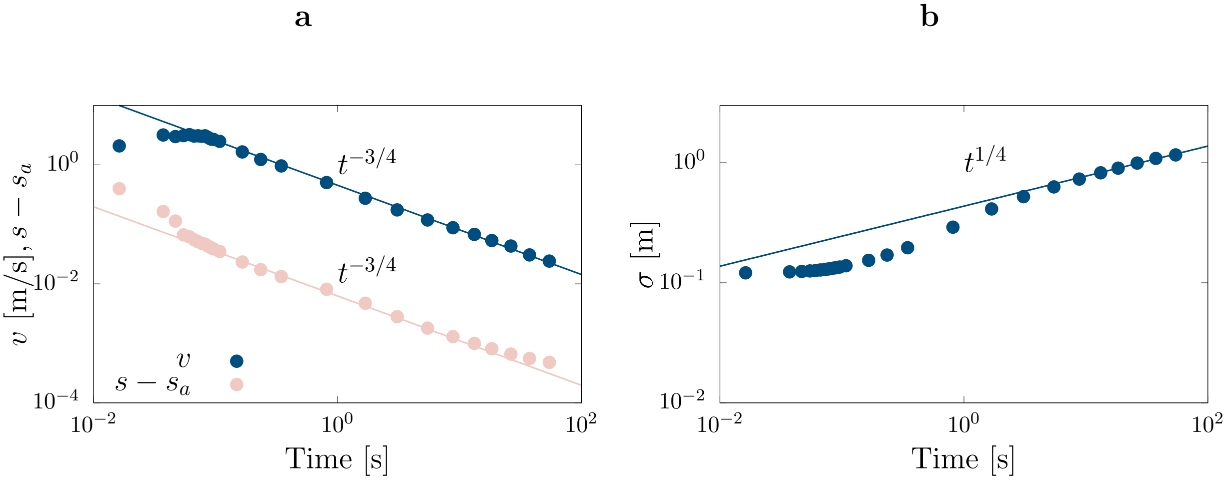

FIG. Supplementary 1. **Validation of turbulent puff dynamics and tracers.** **a** Time history of the mean streamwise velocity component v (blue) and of the supersaturation field $s - s_a$ (pink). The symbols refer to the results from our direct numerical simulations. The lines show the expected scaling for both the velocity and supersaturation field [45]. **b** The standard deviation of a cloud of tracers as a function of time. Symbols refer to the results from our direct numerical simulations; the continuous line is the expected scaling law obtained on the basis of the phenomenological arguments reported in Methods.

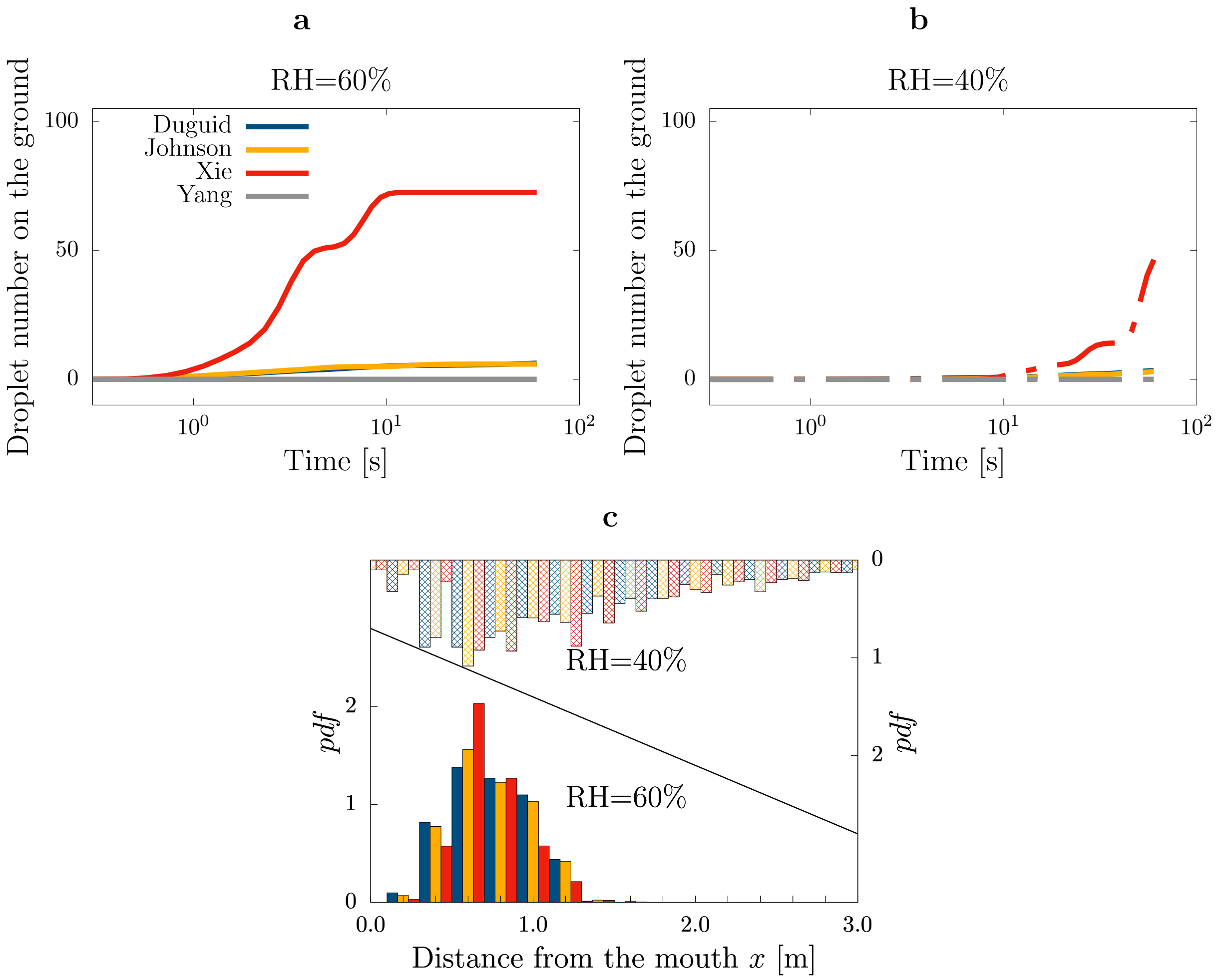

FIG. Supplementary 2. **Sedimentation of large droplets.** **a** Normalized number of droplets settling to the ground, obtained with the four different initial droplet size distributions proposed by Duguid [11] (blue), Johnson *et al.* [16] (yellow), Xie *et al.* [14] (red) and Yang *et al.* [15] (gray). Here, the ambient relative humidity is RH=60%. **b** Same as (a), for a dry environment, RH=40%. **c** Probability density function of the distance from the mouth when droplets reach the ground obtained for all droplet size distributions; environmental relative humidity RH = 60% (solid) and RH = 40% (patterned). Note that, no droplets sediment with the distribution by Yang.

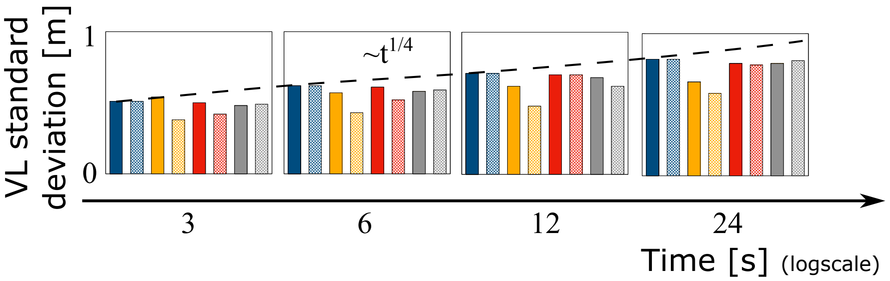

FIG. Supplementary 3. **Airborne-transmitted droplets.** Time evolution of the viral load standard deviation for the eight numerical experiments performed. The dashed line represents the expected power-law growth predicted by means of phenomenological arguments (see Methods). The variability observed for the standard deviations associated to different initial droplet size distributions reaches values of about 30%.

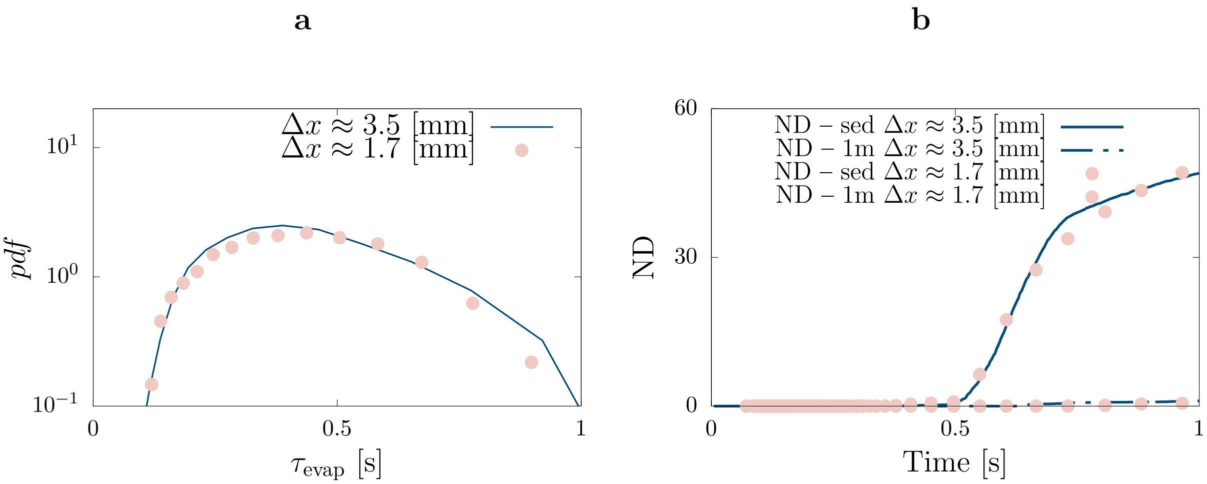

FIG. Supplementary 4. **Grid convergence analysis.** Comparison between results computed with grid spacing $\Delta x = 3.5\,\mathrm{mm}$ (blue lines) and $\Delta x = 1.75\,\mathrm{mm}$ (pink symbols). **a** Probability density function of the particle evaporation time. **b** Cumulative number of sedimenting and airborne droplets (in percentage with respect to the total number of droplets).

SUPPLEMENTARY INFORMATION

The complete list of physical and chemical parameters involved in our model is reported in Table Supplementary 1 along with their baseline values adopted in this investigation. The dry nucleus of droplets is assumed to be composed by a soluble phase (NaCl) and a insoluble phase (mucus). Given the typical value of the mass fraction for the former, the overall density of the dry nucleus can be expressed as

$$\rho_N = \frac{\rho_u}{1 - \epsilon_m [1 - (\rho_u/\rho_s)]} \tag{9}$$

while the density of the entire i-th droplet is

$$\rho_{D\,i} = \rho_w + (\rho_N - \rho_w) \left(\frac{r_{N\,i}}{R_i(t)} \right)^3 , \tag{10}$$

where the radius of the (dry) solid part of the droplet when NaCl is totally crystallized (i.e. below CRH) is given by

$$r_{N\,i} = R_i(0) \left(\frac{\mathcal{C}\,\rho_w}{\mathcal{C}\,\rho_w + \rho_N(1-\mathcal{C})} \right)^{1/3} . \tag{11}$$

Some additional expressions (which can be found in the literature [22, 47] or derived by simple arguments) complete the description:

$$e_{sat} = 6.1078 \times 10^2\, e^{(17.27\,T/(T+237.3))}\,\mathrm{Pa}, \tag{12}$$

$$C_R = \left[\frac{\rho_w\, R_v\, (273.15 + T)}{e_{sat}\, D_v} + \frac{\rho_w\, L_w^2}{k_a\, R_v\, (273.15+T)^2} - \frac{\rho_w\, L_w}{k_a (273.15 + T)} \right]^{-1} , \tag{13}$$

$$A = \frac{2\sigma_w}{R_v (T + 273.15) \rho_w}, \tag{14}$$

$$B = \frac{n_s \Phi_s \epsilon_v M_w \rho_s}{M_s \rho_w}. \tag{15}$$

Here, $n_s = 2$ is the total number of ions into which a salt molecule dissociates, $\Phi_s = 1.2$ is the practical osmotic coefficient of the salt in solution [48] and $\epsilon_v = \epsilon_m(\rho_N/\rho_s)$ is the volume fraction of dry nucleus with respect to the total droplet. Finally, note that in Eqs. (12), (13) and (14) the temperature T is expressed in degrees Celsius.
